# HIF-1α Pulmonary Phenotype Wide Association Study Unveils a Link to Inflammatory Airway Conditions

**DOI:** 10.3389/fgene.2021.756645

**Published:** 2021-09-21

**Authors:** Jelte Kelchtermans, Xiao Chang, Michael E. March, Frank Mentch, Patrick M. A. Sleiman, Hakon Hakonarson

**Affiliations:** ^1^ Perelman School of Medicine, University of Pennsylvania, Philadelphia, PA, United States; ^2^ The Center of Applied Genomics, The Children’s Hospital of Philadelphia, Philadelphia, PA, United States

**Keywords:** phenotype, hypoxia inducible factor, inflammation, airway, HIF1A, rhinitis, bronchiolitis, SNP

## Abstract

Despite experimental data linking HIF-1α dysfunction to inflammatory airway conditions, the effect of single nucleotide polymorphisms within the *HIF1A* gene on these conditions remains poorly understood. In the current study, we complete a phenotype wide association study to assess the link between SNPs with known disease associations and respiratory phenotypes. We report two SNPs of the *HIF1A* gene, the intronic rs79865957 and the missense rs41508050. In these positions the A and the T allele are significantly associated with allergic rhinitis and acute bronchitis and bronchiolitis, respectively. These findings further support the role of HIF-1α in inflammatory pulmonary conditions and may serve as a basis to refine our understanding of other HIF-1α associated phenotypes.

## Introduction

Hypoxia inducible factor (HIF) is central in the mammalian response to hypoxia. (Majmundar et al., 2010) HIF-1 is a nuclear factor that consists of a Hypoxia inducible factor 1α (HIF-1α) and a Hypoxia inducible factor 1β (HIF-1β) subunit. (Wang and Semenza, 1993; Gladek et al., 2017) While HIF-1β is stable regardless of the oxygen concentration, HIF-1α is rapidly degraded under normoxic conditions. (Yu et al., 1998; Gladek et al., 2017) Under hypoxic conditions however, HIF-1α is stabilized, leading to formation of HIF-1. (Ke and Costa, 2006; Slemc and Kunej, 2016) HIF-1 then in turn acts as a transcription factor, affecting over 98 target genes associated with up to 20 biological pathways. (Ke and Costa, 2006; Slemc and Kunej, 2016) Given this central role, it comes as no surprise that variations within the highly conserved *HIF1A* gene have been associated with a wide array of pathologic conditions. (Majmundar et al., 2010) Apart from playing an important role in normal lung development, HIFs have been shown to play a central role in the development of multiple pulmonary conditions, including pulmonary hypertension, Chronic obstructive pulmonary disease (COPD) and lung cancer angiogenesis. (Shimoda and Semenza, 2011) Despite this, within pulmonology, to date, variations within the *HIF1A* gene have only been associated with COPD and lung cancer. (Chan et al., 2017; Gladek et al., 2017; Paradowska-Gorycka et al., 2018; Wang et al., 2018; Hoang et al., 2019; Huang et al., 2020) Our current study sets out to examine the association between single nucleotide polymorphisms (SNPs) in the *HIF1A* gene and respiratory phenotypes. By starting with SNPs of interest, the Phenotype Wide Association Study (PheWAS) design flips the direction of inference commonly used in genome-wide association studies (GWAS). (Bush et al., 2016) To do so, it integrates data captured from patient’s electronic health records (EHRs) with their genetic information. The major benefit of this approach is that it allows us to focus our efforts specifically on SNPs with known disease associations within this master regulator gene, improving the likelihood that found associations are based on molecular mechanisms that are relevant to the disease phenotypes uncovered.

## Methods

### Single Nucleotide Polymorphisms Selection

SNPs were selected from enriched literature review, including recently completed review by Gladek et al. (Gladek et al., 2017) All studies identified with keywords “HIF1a” and “variant” published after their literature review was completed, were reviewed. SNPs significantly associated with human disease were included in the current study (Table 1).

**TABLE 1:** Minor allele frequency for all SNPs included in the current study, unless otherwise indicated the data was pulled from the genome aggregation database (Karczewski et al., 2020).

SNP ID	Substitution	MAF
rs1957757	T>C	0.326–0.952
rs12434438	G>A	0.117–0.841^a^
rs10873142	C>T	0.338–0.914
rs41508050	C>T	0.000292–0.0189
rs2301113	C>A	0.188–0.881
rs11549465	C>T	0.0366–0.155
rs11549467	G>A	0.000574–0.0431
rs199775054	G>C	0.000–0.001^b^
rs113182457rs60361955	insGT	Unavailable
rs2057482	T>C	0.658–0.939
rs2783778	C>T	0.184–0.870
rs7148720	T>C	0.00575–0.151
rs1535679	A>C	0.185–0.872
rs28708675	A>T	0.000218–0.370
rs1319462	G>A	0.682–0.944
rs1957755	G>A	0.000–0.075
rs41362550	T>C	0.0283–0.0755
rs7143164	G>C	0.0485–0.523
rs1951795	A>C	0.227–0.913
rs12435848	A>G	0.249–0.912
rs2301104	G>C	0.000344–0.0147
rs10129270	G>A	0.0311–0.373
rs8005745	T>A	0.369–0.952
rs779897997	C>A	Unavailable
rs4899056	T>C	0.262–0.948
rs11158358	G>C	0.655–0.928
rs2301111	G>C	0.222–0.902
rs966824	T>C	0.717–0.977
rs41492849	C>T	0.0000648–0.00207
rs34005929	G>A	0.000459–0.00943
rs61755645	A>T	0.00161–0.0150
rs4902080	T>C	0.654–0.977
rs4902082	C>T	0.226–0.862
rs17099207	G>A	0.237–0.399
rs142179458	G>A	0.000574–0.0282
rs12434439	G>C	0.110–0.506
rs76308410	C>T	0.0621–0.149
rs74481028	A>G	0.0759–0.226
rs7161527	T>C	0.686–0.939
rs10147275	T>G	0.680–0.939
rs2301108	A>G	0.379–0.952
rs79865957	G>A	0.000230–0.00210

a Data from the Allele Frequency Aggregator (Phan Yj et al., 2020).

b Data from the 1000 Genomes Project (Auton et al., 2015).

### Population

Subjects were drawn from The Children’s Hospital of Philadelphia (CHOP) biorepository at the Center for Applied Genomics (CAG). The pediatric samples included in this biorepository are linked to subjects’ EMRs. All subjects have consented to both genomic analysis and EMR mining (Gottesman et al., 2013).

### Genotype Imputation

Genotype data were generated by the Center for Applied Genomics on patients recruited from CHOP and were acquired on four major genotyping arrays (HumanHapMap550, 610Q, OMNI2.5M and the GSA array). Where possible, data from similar arrays were merged. Data were filtered for genotype missingness (geno 0.1), individual missingness (0.02), and minor allele frequency (MAF) (0.01) using PLINK v1.9. (Chang et al., 2015) Data were imputed using the TOPMed v2 reference panel on the TOPMed Imputation Server. (Fuchsberger et al., 2015; Das et al., 2016; Taliun et al., 2021) Imputed genotypes were filtered on combinations of Rsq (imputation quality metric) and MAF [(MAF ≥ 0.05 and Rsq > 0.3) OR (MAF < 0.05 and Rsq > 0.5)] using BCFTools v1.10.2, and only SNPs that remained in 85% of samples were retained for use in PheWAS analysis (Danecek et al., 2021).

### Ancestry Identification

Subjects in the PheWAS cohort were separated by ancestry based on the results of principal component analysis (PCA). PCA was performed using flashpca on approximately 2.4 million imputed SNPs with MAF >0.01 that had been pruned for linkage disequilibrium using PLINK v1.9 (Abraham and Inouye, 2014; Chang et al., 2015) The first three principle components were plotted, and ancestry designation was performed by comparison to the reference genotypes from the HapMap consortium. (Altshuler et al., 2010) The complete dataset contained 71,600 individuals: 34,410 Caucasians, 31,507 African Americans, 2644 Hispanics, and 3039 East Asians.

### PheWAS

A PheWAS was conducting using the published PheWAS R package from Carroll et al. (v0.99.5-5). (Carroll et al., 2014) International Classification of Diseases 9 (ICD-9) codes were obtained from an anonymized extraction of the Children’s Hospital of Philadelphia diagnosis database that contained subjects that had been recruited into the patient collection of the Center for Applied Genomics. Counts of the occurrence of each ICD-9 code for each subject were generated, and the resulting table was converted into the PheWAS phenotype table by a function in the R package. Subjects were included in the case group for each PheWAS phenotype if they possessed two or more occurrences of any of the ICD-9 codes that composed the phenotype in question. Subjects were listed as controls for the PheWAS phenotype if they lacked the case-defining ICD-9 codes, as well as ICD-9 codes corresponding to closely related phenotypes. Conversion from ICD-9 codes to PheWAS phenotypes was performed using the default translation table included in the R package. Phenotypes were analyzed in the PheWAS if they were represented by 20 or more cases in the cohort. The subject’s sex and age were included as covariates in the analysis, as were the 10 flashpca generated principle components and a variable representing the group in which genotyping array had been imputed. Genotypes were extracted from the imputed data as allele dose information to preserve some information regarding genotype probability, and the allele doses were used as the genotype inputs to the PheWAS. The PheWAS analysis was performed individually on each PCA-defined ancestry, and then a meta-analysis was performed combining all four ancestries using the PheWAS-meta function provided in the PheWAS R package. For the association test, a logistic regression model, adjusted for age and sex was used. For defining significance in this study, we set a FDR threshold of 0.05. As a total of 2146 traits were analyzed, the over-conservative significance threshold based on Bonferroni correction was *p* = 2.3 × 10^–5^.

### In Silico Validation

SNP’s significantly associated with respiratory disease were validated in an independent cohort by querying the publicly available Open Target Genetics database. (Ghoussaini et al., 2021) The Ensembl VEP was then used to assess the likely effect of these variants. (McLaren et al., 2016) To assess chromatin state and regulatory potential associated with the locations of the SNPs, other publicly available databases including Haploreg and Encode were queried.

## Results

We found 42 SNPs that have been previously associated with different medical conditions, including various cancers, cardiovascular diseases, metabolic disorders and (auto) immune diseases. This includes the 34 SNPs identified by Gladek et al. (Gladek et al., 2017) In addition, eight more SNPs were identified in studies published after their literature review was completed (Chan et al., 2017; Paradowska-Gorycka et al., 2018; Wang et al., 2018; Hoang et al., 2019; Huang et al., 2020).

**FIGURE 1:**
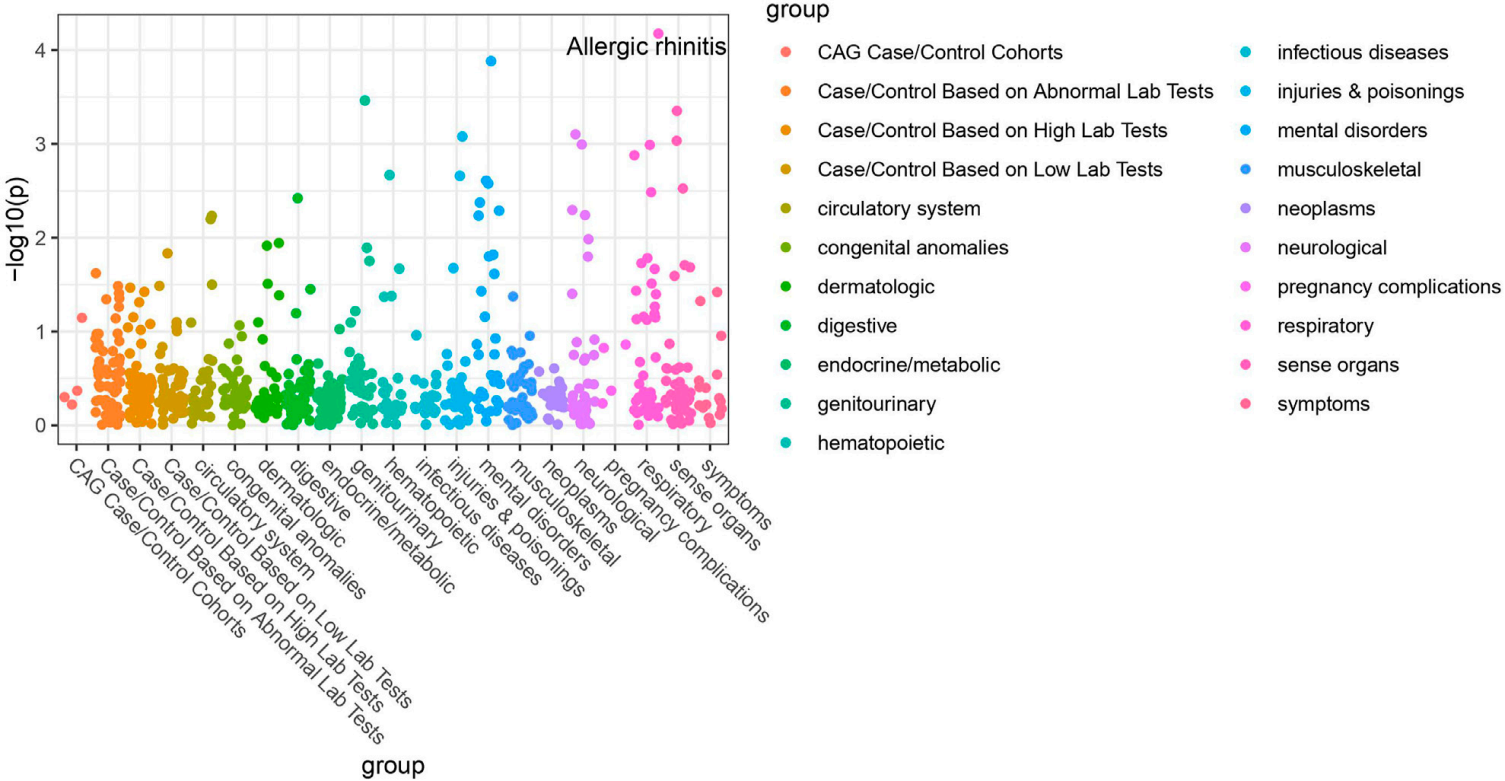
PheWAS results for rs79865957.

**FIGURE 2:**
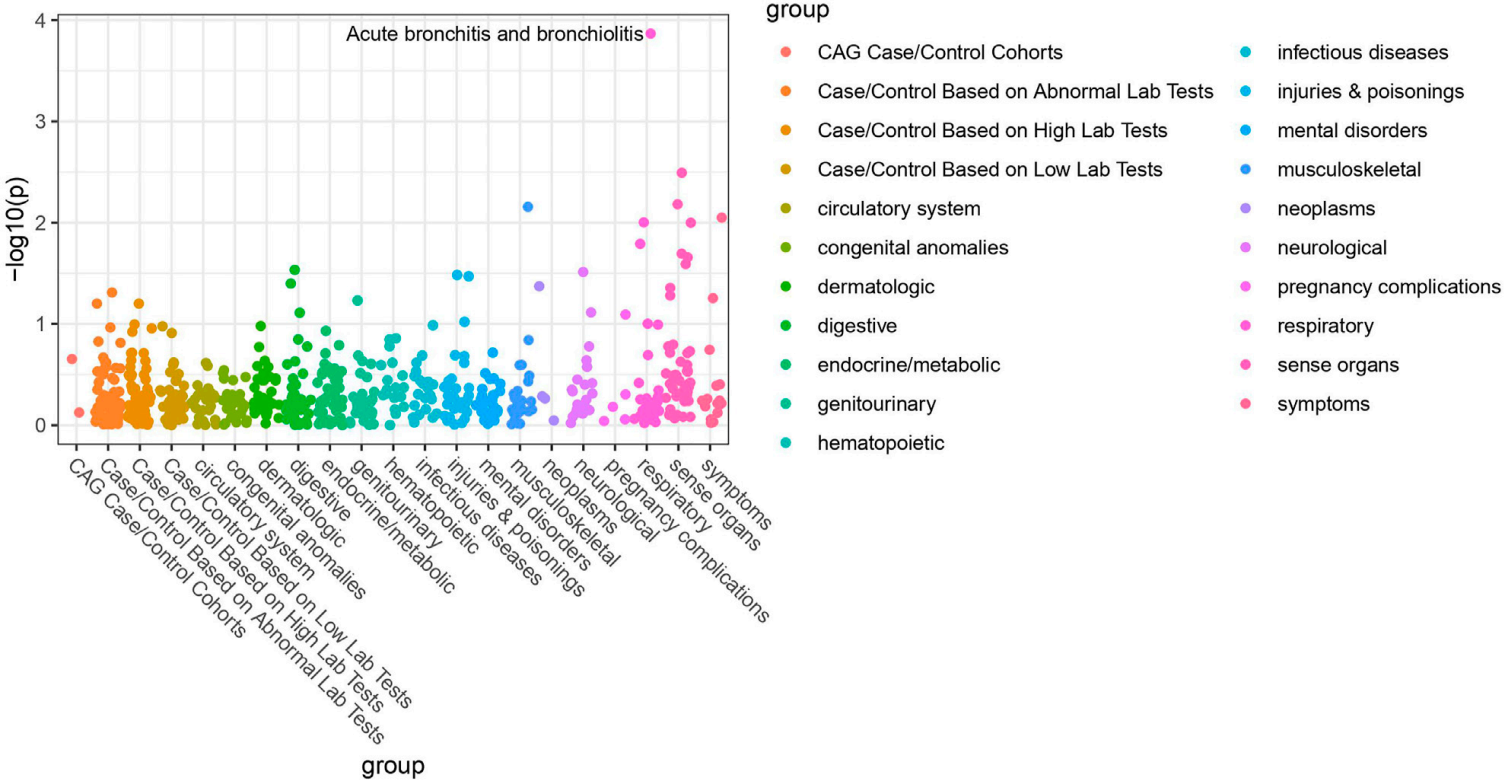
PheWAS results for rs41508050.

Of the 42 SNPs included in our PheWAS, nine were significantly associated with at least one disorder. Table 2 summarizes the data for all the SNP-phenotype associations passing False Discovery Rate (FDR) or Bonferroni test. Most of the detected associations were from cohorts with less than 500 cases. However, the A allele of SNP rs79865957 was found to be significantly associated with allergic rhinitis (Figure 1) in a European cohort of 4,348 cases and 18,794 controls with an allele frequency of 0.08%. The OR was 2.86, Beta 1.05, SE 0.25 and *p*-value 3.48E−05. The second, rs41508050, the T allele was significantly associated with acute bronchitis and bronchiolitis (Figure 2) in an African American cohort of 2,234 cases and 21,463 controls with an allele frequency of 0.18%. The OR was 0.32, Beta 1.21, SE 3.36 and *p*-value 0.0001.

**TABLE 2:** SNP-phenotype associations passing False Discovery Rate (FDR) or Bonferroni test.

Population	phecode	Description	snp	beta	OR	SE	P	n_cases	n_controls	bonferroni	fdr
European	573.3	Hepatomegaly	rs61755645	1.728033876	0.409801308	5.629574578	2.48E−05	107	30,884	TRUE	TRUE
	251.1	Hypoglycemia	rs61755645	1.33952494	0.337650972	3.817229663	7.27E−05	264	28,603	FALSE	TRUE
	476	Allergic rhinitis	rs79865957	1.111814428	0.278793888	3.039869017	6.66E−05	4348	18,794	FALSE	TRUE
	155.1	Malignant neoplasm of liver, primary	rs142179458	2.97285E+15	86170822.24	Inf	0	20	32,275	TRUE	TRUE
	961.1	Poisoning/allergy of sulfonamides	rs142179458	74.05386998	17.24313765	1.45E+32	1.75E−05	118	28,063	TRUE	TRUE
	696	Psoriasis and related disorders	rs28708675	1.346806715	0.340112245	3.845127319	7.50E−05	220	29,124	FALSE	TRUE
African	242	Thyrotoxicosis with or without goiter	rs142179458	3.491251843	0.848041087	32.82701628	3.84E−05	72	28,558	TRUE	TRUE
	569	Other disorders of intestine	rs142179458	3.29947623	0.80271968	27.09844186	3.95E−05	97	28,861	TRUE	TRUE
	531	Peptic ulcer (excl. esophageal)	rs142179458	4.401347385	1.148701204	81.56068832	0.00012732	20	29,609	FALSE	TRUE
	578.9	Hemorrhage of gastrointestinal tract	rs142179458	4.169090079	1.137501266	64.65659292	0.000247213	31	27,843	FALSE	TRUE
	427.11	Paroxysmal supraventricular tachycardia	rs142179458	3.94818387	1.106947085	51.84113103	0.00036146	38	26,415	FALSE	TRUE
	300.9	Posttraumatic stress disorder	rs1951795	0.671463911	0.162919352	1.957100245	3.76E−05	91	23,629	TRUE	TRUE
	300.9	Posttraumatic stress disorder	rs2301111	0.720505023	0.162403177	2.055471008	9.14E−06	91	23,629	TRUE	TRUE
	379.2	Disorders of vitreous body	rs34005929	5.024227052	1.198711093	152.0526818	2.77E−05	21	25,418	TRUE	TRUE
	573.5	Jaundice (not of newborn)	rs34005929	3.027318192	0.742782183	20.64180095	4.59E−05	235	28,047	FALSE	TRUE
	483	Acute bronchitis and bronchiolitis	rs41508050	1.211842424	0.317605334	3.359668887	0.000135874	2234	21,463	FALSE	TRUE
	327.7	Sleep related movement disorders	rs79865957	4.791384583	1.207932763	120.4680513	7.29E−05	35	23,361	FALSE	TRUE
Asian	195	Cancer, suspected or other	rs2301104	2.851754693	0.671497479	17.31814322	2.17E−05	25	2616	TRUE	TRUE
	348	Other conditions of brain	rs2301104	1.929317425	0.492152257	6.884809237	8.85E−05	51	2399	TRUE	TRUE
	801.1	Fracture of foot	rs79865957	−3.2555E+14	52249787.97	0	0	20	2474	TRUE	TRUE

Using the Open Target Genetics database rs79865957, the A allele was found to have been previously positively associated with both chronic airway obstruction (OR 1.94, *p*-value 0.0019, Beta 0.663) and asthma (OR 1.34, *p*-value 0.033, Beta 0.292). It has also been negatively associated with paternal chronic bronchitis/emphysema (OR 0.75, *p*-value 0.0069, Beta −0.293). Using Ensemble Variable Effect Predictor (VEP), it was found to be a likely intron variant for *HIF1Α*. For rs41508050, the T allele was previously negatively correlated with “Bring up phlegm/sputum/mucus on most days” (OR 0.72, *p*-value 0.0026, Beta −0.328) and is a missense variant for *HIF1Α*.

The publicly available HaploReg tool was queried for both SNPs. SNP rs79865957 has four SNPs in linkage disequilibrium (r2 ≥ 0.8), two of which (rs76269977 and rs142660658) are intronic in the *HIF1A* gene. It is located in a regulatory region but not in a constrained sequence. It has histone H3K4me1_Enh enhancer marks in a lung carcinoma line and both H3K4me1_Enh and H3K27ac in a fetal lung fibroblast line. It is also a DNAse hypersensitivity site in a fetal lung fibroblast line. SNP rs41508050 has no other SNPs in linkage disequilibrium, is in a regulatory region and in a constrained sequence both by Genomic Evolutionary Rate Profiling and SiPhycons. It has histone H3K27ac_Enh marks in both lung fibroblast and lung carcinoma lines and is a DNase hypersensitivity site in a lung carcinoma cell line. Looking at Encode it had RFX5 bound in the GM12878 lymphoblastoid cell line. (Table 3)

**TABLE 3:** Summary of chromatin state and regulatory potential associated with the locations of the SNPs.

Location	rs79865957	rs41508050
Reference allele	G	C
Varriant allele	A	T
Regulatory region?	Yes	Yes
Constrained sequence?	No	Yes
DNAse hypersensitivity site?	In fetal fibroblast line	In lung carcinoma cell line
Associated Histone markers	H3K4me1_Enh and H3K27ac	H3K27ac_Enh
Encode	No proteins bound	RFX5 bound

## Discussion

We present the results of a HIF-1α PheWAS analysis focused on association with respiratory phenotypes. We identified two SNPs that are significantly associated with respiratory disease. Given the allele rarity in our patient population, the Open Target Genetics database was queried in further support. This resource integrates knowledge derived from the UK Biobank with published data from other sources and provides an independent cohort to validate our findings. (Baumann and Cabassa, 2020) The prior associations with allergic airway disease in the form of asthma for rs79865957 and association with bringing up phlegm/sputum/mucus for rs41508050 are consistent with the respective associations with allergic rhinitis and acute bronchitis and bronchiolitis in our cohort, suggesting the association may be driven by the underlying biological “inflammation” process which is the central driver across all these phenotypes involving different organs. To address the likely impact of these variants we used the Ensembl VEP and the publicly available HaploReg tool (Ward and Kellis, 2012; McLaren et al., 2016), both of which underscore the possible significance of both variants. Adding to the evidence supporting a functional impact are the previously published associations between rs79865957 and diabetic kidney disease and between rs41508050 and angina versus myocardial infarction as initial presentation of coronary disease (Hlatky et al., 2007; Huang et al., 2020).

Previously, variations within the *HIF1A* gene have been associated with COPD, lung cancer and a host of non-pulmonary conditions. (Gladek et al., 2017) Both the SNPs reported here had prior significant disease associations. First, rs79865957 was previously associated with diabetic kidney disease in a Han Chinese population. (Nava-Salazar et al., 2011) While to our knowledge the functional consequences of this SNP have not been eluded, the authors hypothesized that in a high glucose environment *HIF1A* transcription may be stimulated. Additionally, rs41508050 has a known association with the development of stable angina as opposed to myocardial infarction as initial presentation of coronary artery disease. (Hlatky et al., 2007) *In vitro* studies have previously linked this variant with a higher transcriptional activity. (Nava-Salazar et al., 2011) However, to our knowledge, the current study is the first to report on the association between SNPs of the *HIF1A* gene and allergic rhinitis, acute bronchitis and bronchiolitis. The reported association with allergic rhinitis is consistent with previously published experimental data highlighting the role of HIF-1α in allergic airway pathology. In an allergic airway disease model, HIF-1α inhibition decreased Th2 inflammation as measured by reduced IL-4, IL-5 and IL-13. (Kim et al., 2010) Beyond this, in a mouse model downregulation of HIF-1 or blockade of HIF-1α reduced cellular infiltrate in peribronchial lung tissues, thickness of smooth muscle and eosinophil infiltration. (Huerta-Yepez et al., 2008) Likewise, the role of HIF-1α in bronchiolitis is supported by experimental data on the consequences of HIF-1α stabilization by the Respiratory Syncytium Virus. (Kilani et al., 2004)

Traditionally, GWAS identify SNPs significantly associated with human disease. These findings are then used to guide animal studies aiming to prove a causal link between the SNP and the disease. As briefly discussed above, the PheWAS design flips this process. It allowed us to look specifically at a highly conserved gene known to play a central role in the diseases of interest. In doing so, we were able to narrow down the list of SNPs within the *HIF1A* gene that play a potential role in respiratory pathology. Beyond this, we were able to detect significant effects of rare allelic variants. Conversely, this study design by definition excludes variants on other genes. While this is a limitation of the current study, given the hypoxemia dependent stabilization of HIF-1α and the experimental data supporting a role of HIF-1α in pulmonary conditions as outlined above it seemed reasonable to focus on *HIF1A*. Future studies may expand on the current work by including other members of the HIF family. Furthermoree, knowing that SNPs within the *HIF1A* gene are associated with respiratory diseases future studies can now refine our understanding of the associated phenotypes by looking at differences between patients with and without these SNPs.

## Data Availability

The data analyzed in this study is subject to the following licenses/restrictions: Some of the data used are available in deidentified format in dbGaP. Requests to access these datasets should be directed to https://www.ncbi.nlm.nih.gov/gap/.

## References

[B1] Abraham G. Inouye M. (2014). Fast Principal Component Analysis of Large-Scale Genome-wide Data. PloS one 9 (4), e93766. 10.1371/journal.pone.0093766 24718290 PMC3981753

[B2] Altshuler D. M. Gibbs R. A. Peltonen L. Altshuler D. M. Gibbs R. A. Peltonen L. (2010). Integrating Common and Rare Genetic Variation in Diverse Human Populations. Nature 467 (7311), 52–58. 10.1038/nature09298 20811451 PMC3173859

[B3] Auton A. Auton A. Brooks L. D. Durbin R. M. Garrison E. P. Kang H. M. (2015). A Global Reference for Human Genetic Variation. Nature 526 (7571), 68–74. 10.1038/nature15393 26432245 PMC4750478

[B4] Baumann A. A. Cabassa L. J. (2020). Reframing Implementation Science to Address Inequities in Healthcare Delivery. BMC Health Serv. Res. 20 (1), 190. 10.1186/s12913-020-4975-3 32164706 PMC7069050

[B5] Bush W. S. Oetjens M. T. Crawford D. C. (2016). Unravelling the Human Genome-Phenome Relationship Using Phenome-wide Association Studies. Nat. Rev. Genet. 17 (3), 129–145. 10.1038/nrg.2015.36 26875678

[B6] Carroll R. J. Bastarache L. Denny J. C., and (2014). R PheWAS: Data Analysis and Plotting Tools for Phenome-wide Association Studies in the R Environment. Bioinformatics 30 (16), 2375–2376. 10.1093/bioinformatics/btu197 24733291 PMC4133579

[B7] Chan C. H. T. Munusamy P. Loke S. Y. Koh G. L. Wong E. S. Y. Law H. Y. (2017). Identification of Novel Breast Cancer Risk Loci. Cancer Res. 77 (19), 5428–5437. 10.1158/0008-5472.can-17-0992 28775167

[B8] Chang C. C. Chow C. C. Tellier L. C. Vattikuti S. Purcell S. M. Lee J. J. (2015). Second-generation PLINK: Rising to the Challenge of Larger and Richer Datasets. GigaScience 4, 7. 10.1186/s13742-015-0047-8 25722852 PMC4342193

[B9] Danecek P. Bonfield J. K. Liddle J. Marshall J. Ohan V. Pollard M. O. (2021). Twelve Years of SAMtools and BCFtools. GigaScience 10 (2), giab008. 10.1093/gigascience/giab008 33590861 PMC7931819

[B10] Das S. Forer L. Schönherr S. Sidore C. Locke A. E. Kwong A. (2016). Next-generation Genotype Imputation Service and Methods. Nat. Genet. 48 (10), 1284–1287. 10.1038/ng.3656 27571263 PMC5157836

[B11] Fuchsberger C. Abecasis G. R. Hinds D. A. (2015). Minimac2: Faster Genotype Imputation. Bioinformatics 31 (5), 782–784. 10.1093/bioinformatics/btu704 25338720 PMC4341061

[B12] Ghoussaini M. Mountjoy E. Carmona M. Peat G. Schmidt E. M. Hercules A. (2021). Open Targets Genetics: Systematic Identification of Trait-Associated Genes Using Large-Scale Genetics and Functional Genomics. Nucleic Acids Res. 49 (D1), D1311–D1320. 10.1093/nar/gkaa840 33045747 PMC7778936

[B13] Gladek I. Ferdin J. Horvat S. Calin G. A. Kunej T. (2017). HIF1Agene Polymorphisms and Human Diseases: Graphical Review of 97 Association Studies. Genes Chromosom. Cancer 56 (6), 439–452. 10.1002/gcc.22449 28165644 PMC5395341

[B14] Gottesman O. Kuivaniemi H. Kuivaniemi H. Tromp G. Faucett W. A. Li R. (2013). The Electronic Medical Records and Genomics (eMERGE) Network: Past, Present, and Future. Genet. Med. 15 (10), 761–771. 10.1038/gim.2013.72 23743551 PMC3795928

[B15] Hlatky M. A. Quertermous T. Boothroyd D. B. Priest J. R. Glassford A. J. Myers R. M. (2007). Polymorphisms in Hypoxia Inducible Factor 1 and the Initial Clinical Presentation of Coronary Disease. Am. Heart J. 154 (6), 1035–1042. 10.1016/j.ahj.2007.07.042 18035072

[B16] Hoang T. T. Manso P. H. Edman S. Mercer-Rosa L. Mitchell L. E. Sewda A. (2019). Genetic Variants of HIF1α Are Associated with Right Ventricular Fibrotic Load in Repaired Tetralogy of Fallot Patients: A Cardiovascular Magnetic Resonance Study. J. Cardiovasc. Magn. Reson. 21 (1), 51. 10.1186/s12968-019-0555-2 31422771 PMC6699069

[B17] Huang Y. Jin L. Yu H. Jiang G. Tam C. H. T. Jiang S. (2020). SNPs in PRKCA-Hif1a-GLUT1 Are Associated with Diabetic Kidney Disease in a Chinese Han Population with Type 2 Diabetes. Eur. J. Clin. Invest.,50 (9), e13264. 10.1111/eci.13264 32394523

[B18] Huerta-Yepez S. Baay-Guzman G. J. Garcia-Zepeda R. Hernandez-Pando R. Vega M. I. Gonzalez-Bonilla C. (2008). 2-Methoxyestradiol (2-ME) Reduces the Airway Inflammation and Remodeling in an Experimental Mouse Model. Clin. Immunol. 129 (2), 313–324. 10.1016/j.clim.2008.07.023 18793875

[B19] Karczewski K. J. Francioli L. C. Tiao G. Cummings B. B. Alföldi J. Wang Q. (2020). The Mutational Constraint Spectrum Quantified From Variation in 141,456 Humans. Nature 581 (7809), 434–443. 10.1038/s41586-020-2308-7 32461654 PMC7334197

[B20] Ke Q. Costa M. (2006). Hypoxia-inducible Factor-1 (HIF-1). Mol. Pharmacol. 70 (5), 1469–1480. 10.1124/mol.106.027029 16887934

[B21] Kilani M. M. Mohammed K. A. Nasreen N. Tepper R. S. Antony V. B. (2004). RSV Causes HIF-1α Stabilization via NO Release in Primary Bronchial Epithelial Cells. Inflammation 28 (5), 245–251. 10.1007/s10753-004-6047-y 16133997

[B22] Kim S. R. Lee K. S. Park H. S. Park S. J. Min K. H. Moon H. (2010). HIF-1α Inhibition Ameliorates an Allergic Airway Disease via VEGF Suppression in Bronchial Epithelium. Eur. J. Immunol. 40 (10), 2858–2869. 10.1002/eji.200939948 20827786

[B23] Majmundar A. J. Wong W. J. Simon M. C. (2010). Hypoxia-inducible Factors and the Response to Hypoxic Stress. Mol. Cel. 40 (2), 294–309. 10.1016/j.molcel.2010.09.022 PMC3143508 20965423

[B24] McLaren W. Gil L. Hunt S. E. Riat H. S. Ritchie G. R. S. Thormann A. (2016). The Ensembl Variant Effect Predictor. Genome Biol. 17 (1), 122. 10.1186/s13059-016-0974-4 27268795 PMC4893825

[B25] Nava-Salazar S. Sánchez-Rodríguez E. N. Mendoza-Rodríguez C. A. Moran C. Romero-Arauz J. F. Cerbón M. A. (2011). Polymorphisms in the Hypoxia-Inducible Factor 1 Alpha Gene in Mexican Patients with Preeclampsia: A Case-Control Study. BMC Res. Notes 4 (1), 68. 10.1186/1756-0500-4-68 21414224 PMC3076269

[B26] Paradowska-Gorycka A. Stypinska B. Pawlik A. Haladyj E. Romanowska-Próchnicka K. Olesinska M. (2018). HIF-1A Gene Polymorphisms and its Protein Level in Patients with Rheumatoid Arthritis: a Case-Control Study. Inflamm. Res. 67 (5), 423–433. 10.1007/s00011-018-1134-y 29411043

[B27] Phan Yj L. Zhang H. Qiang W. Shekhtman E. Shao D. Revoe D. (2020). ALFA: Allele Frequency Aggregator. Bethesda, MD: National Center for Biotechnology Information, US National Library of Medicine.

[B28] Shimoda L. A. Semenza G. L. (2011). HIF and the Lung. Am. J. Respir. Crit. Care Med. 183 (2), 152–156. 10.1164/rccm.201009-1393pp 21242594 PMC3159088

[B29] Slemc L. Kunej T. (2016). Transcription Factor HIF1A: Downstream Targets, Associated Pathways, Polymorphic Hypoxia Response Element (HRE) Sites, and Initiative for Standardization of Reporting in Scientific Literature. Tumor Biol. 37 (11), 14851–14861. 10.1007/s13277-016-5331-4 27644243

[B30] Taliun D. Harris D. N. Kessler M. D. Carlson J. Szpiech Z. A. Torres R. (2021). Sequencing of 53,831 Diverse Genomes from the NHLBI TOPMed Program. Nature 590 (7845), 290–299. 10.1038/s41586-021-03205-y 33568819 PMC7875770

[B31] Wang D. Fan Y. Malhi M. Bi R. Wu Y. Xu M. (2018). Missense Variants in HIF1A and LACC1 Contribute to Leprosy Risk in Han Chinese. Am. J. Hum. Genet. 102 (5), 794–805. 10.1016/j.ajhg.2018.03.006 29706348 PMC5986702

[B32] Wang G. L. Semenza G. L. (1993). General Involvement of Hypoxia-Inducible Factor 1 in Transcriptional Response to Hypoxia. Proc. Natl. Acad. Sci. 90 (9), 4304–4308. 10.1073/pnas.90.9.4304 8387214 PMC46495

[B33] Ward L. D. Kellis M. (2012). HaploReg: A Resource for Exploring Chromatin States, Conservation, and Regulatory Motif Alterations Within Sets of Genetically Linked Variants. Nucleic Acids Res. 40 (Database issue), D930–D934. 10.1093/nar/gkr917 22064851 PMC3245002

[B34] Yu A. Y. Frid M. G. Shimoda L. A. Wiener C. M. Stenmark K. Semenza G. L. (1998). Temporal, Spatial, and Oxygen-Regulated Expression of Hypoxia-Inducible Factor-1 in the Lung. Am. J. Physiol. Lung Cell Mol. Physiol. 275 (4), L818–L826. 10.1152/ajplung.1998.275.4.l818 9755115

